# Population genetic structure of *Schistosoma bovis* in Cameroon

**DOI:** 10.1186/s13071-019-3307-0

**Published:** 2019-01-24

**Authors:** Félicité Flore Djuikwo-Teukeng, Alain Kouam Simo, Jean-François Allienne, Olivier Rey, Arouna Njayou Ngapagna, Louis Albert Tchuem-Tchuente, Jérôme Boissier

**Affiliations:** 1 Université des Montagnes, Faculty of Heath Science, PO Box: 208, Bangangté, Cameroon; 2 Université de Perpignan Via Domitia, IHPE UMR 5244, CNRS, IFREMER, Université de Montpellier, F-66860 Perpignan, France; 3 Centre for Schistosomiasis and Parasitology, Yaoundé, Cameroon; 4 Laboratory of Parasitology and Ecology, Faculty of Sciences, University of Yaoundé I, Yaoundé, Cameroon

**Keywords:** *Schistosoma bovis*, Barcoding, Population genetic structure

## Abstract

**Background:**

Schistosomiasis is neglected tropical parasitic disease affecting both humans and animals. Due to the human health impact, population genetic studies have focused on the three main human-infecting schistosome species: *Schistosoma mansoni*, *S. haematobium* and *S. japonicum*. Here we present novel data on the population genetic structure of *Schistosoma bovis*, a highly widespread and prevalent schistosome infecting ruminants, and therefore of veterinary importance.

**Methods:**

Adult *S. bovis* were sampled in the two main abattoirs of Cameroon (Yaoundé and Douala). Twenty-two cows originating from four distinct localities were sampled and a total of 218 parasites were recovered. All parasites were genotyped using a panel of 14 microsatellite markers and a sub-sample of 91 parasites were sequenced and characterized with the mitochondrial (*cox*1) and nuclear (ITS) genetic markers.

**Results:**

No significant difference in allelic richness, heterozygosity, nucleotide diversity and haplotype diversity was observed between the populations. Additionally, no strong genetic structure was observed at the country scale. Our data also show that *S. bovis* is more polymorphic than its sister species, *S. haematobium*, and that the haplotype diversity is similar to that of *S. mansoni* while the nucleotide diversity does not significantly differ from that of *S. haematobium*. The resulting negative Tajima’s D* and Fu and Li’s D* indices could be a signature of population demographic expansion. No *S. haematobium*/*S. bovis* hybrids were observed in our populations, thus all samples were considered as pure *S. bovis.*

**Conclusions:**

This study provides novel insights into genetic diversity and population genetic structure of *S. bovis*. No strong genetic structure was observed at the country scale but some genetic indices could be associated as a signature of population demographic expansion.

**Electronic supplementary material:**

The online version of this article (10.1186/s13071-019-3307-0) contains supplementary material, which is available to authorized users.

## Background

Schistosomiasis is a chronic and debilitating water-borne parasitic disease of humans and animals. It poses serious public health and veterinary concerns worldwide particularly in tropical and subtropical areas. In humans, the disease affects nearly 218 million people in 78 countries with an estimated 200,000 deaths per year. Almost 95% of the infected people live in Africa [1]. Six species of schistosome are known to infect humans: *Schistosoma mansoni*, *S. haematobium*, *S. intercalatum* and *S. guineensis* in Africa, and *S. mekongi* and *S. japonicum* in Asia [2]. Animals can be infected by 19 species of schistosomes of which five receive particular attention because of the associated infection severity observed in domestic ruminants in Africa (*S. mattheei*, *S. bovis*, *S. curassoni*) and in Asia (*S. japonicum* and *S. mekongi*) [3]. Regarding livestock infections, it has been estimated that 165 million cattle are infected worldwide [4]. Although human schistosomiasis is well monitored in most countries, bovine schistosomiasis receives negligible attention in comparison. In particular, the repartition, prevalence, intensity and transmission dynamics of livestock infections are poorly documented.

Environmental changes due to natural phenomena or human activities particularly agricultural practices associated with dam construction, can have a significant impact on the epidemiology and distribution of human and animal schistosomiasis and may also lead to interspecies hybridization events [5]. For instance, the creation of the Diama Dam on the Senegal River has created a more stable flow of water, creating irrigation canals, rice-growing areas and water points for animals, leading to the emergence of new human and livestock schistosomiasis outbreaks [6, 7]. In Cameroon, creation of the Lagdo Dam on the Benoue River also increased the prevalence of human schistosomiasis [8] and in the same way, the deforestation of the Loum rainforest allowed the establishment of *B. truncatus*, the intermediate host of *S. haematobium* thus allowing its introgressive hybridization with *S. guineensis* (formerly known as *S. intercalatum*) [9, 10]. Seasonal changes in cattle rearing methods in Africa (transhumance, nomadism) are important in the movement of domestic livestock, so animals can come into contact with multiple potential schistosomiasis transmission sites. Hybrids are known to exist between species that infect humans but also between those that have different hosts such as animals and humans [3]. The latter hybrid forms are particularly worrying because they theoretically could be enabling zoonotic transmission.

In Cameroon, out of 23 million inhabitants, there are five million people at risk of schistosomiasis, of which two million are infected [11] with three schistosome species [*S. haematobium*, *S. mansoni* and *S. guineensis* (formerly known as *S. intercalatum*)] involved in human infections, creating a considerable human health concern [11]. Urogenital schistosomiasis, caused by *S. haematobium*, is endemic in northern Cameroon and other foci are located in the south of the country in Kékem, Loum, Barombi-kotto, Kumba and Marumba [12–14]. Limited data are available concerning bovine schistosomiasis. In 1990, the prevalence of ruminant schistosomiasis in northern Cameroon (Garoua and Maroua cities), was recorded using egg morphological diagnostics, as 79.5 and 10.03% for *S. bovis* and *S. curassoni*, respectively [15]. Since several *Schistosoma* species are endemic to Cameroon, interspecific mating between different species have been reported between *S. haematobium × S. guineensis* and also between *S. haematobium × S. mansoni* [16, 17] but there are no data on *S. haematobium × S. bovis* hybrids. *Schistosoma haematobium* is a human parasite living in the veins around the urinary bladder while *S. bovis* is a livestock parasite living in the mesenteric veins around the gut. *S. haematobium × S. bovis* hybrids are largely present in West Africa, including Senegal, Mali, Benin and Niger [3, 18]. This latter hybrid has also recently emerged in the south of Europe [19–21].

Here, our aim is to document how populations of *S. bovis* are structured in Cameroon at a large geographical scale. We used genetic approaches, including both mitochondrial (*cox*1) and nuclear markers (ITS and microsatellites), first to characterize the population genetic structure of *S. bovis* and secondly to identify potential *S. haematobium × S. bovis* hybrids in Cameroon. Additionally, we took advantage of the fact that the microsatellite markers used in this study are informative for both *S. haematobium* and *S. bovis* to compare the genetic diversity between these two sister species.

## Methods

### Parasite sampling from cattle

**Fig. 1:**
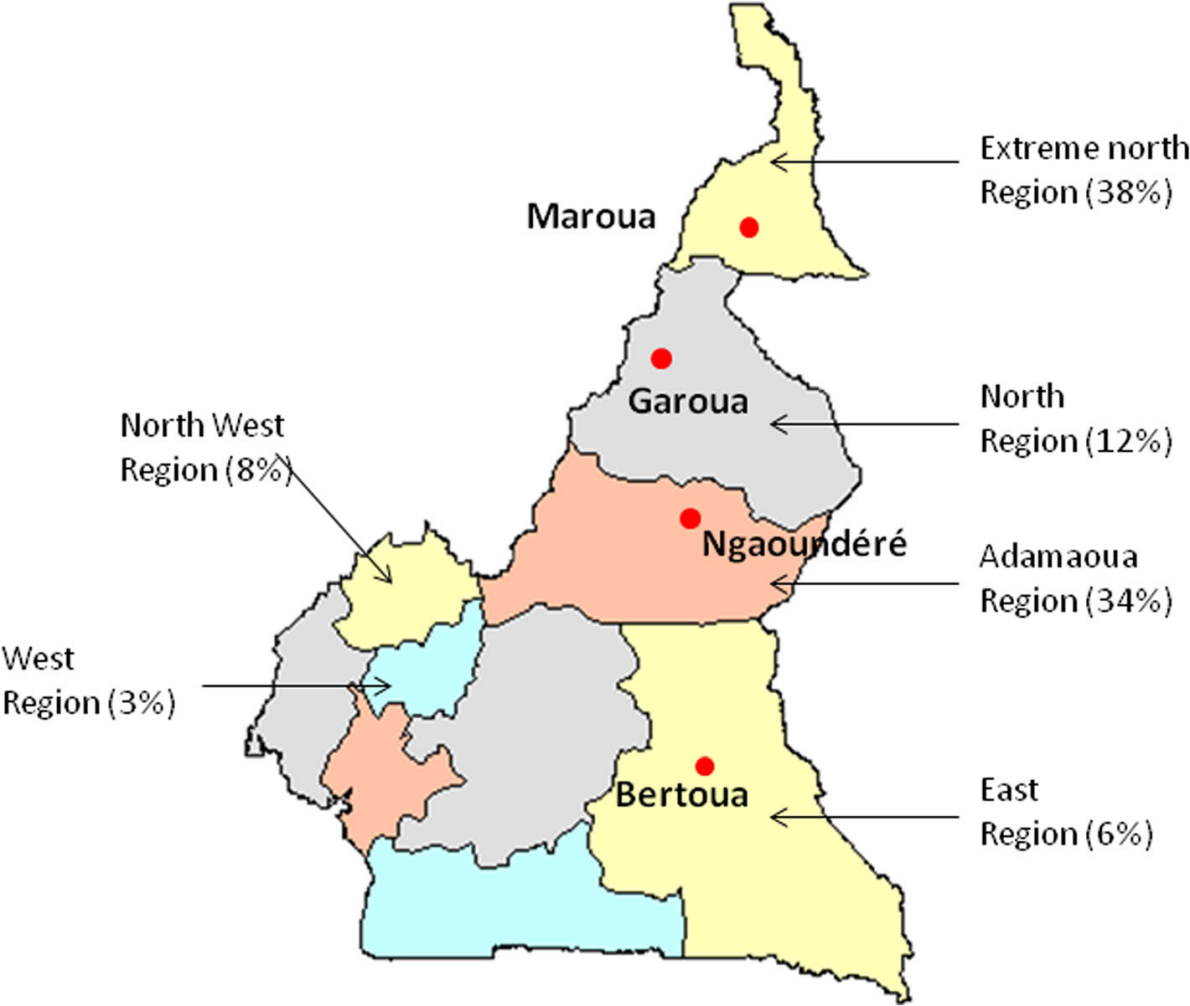
Map of the areas from where the cows originated: Maroua, Garoua, Ngaoundéré and Bertoua. The different proportions of cattle breeding in each area are shown as a percentage [35]

Authorization for parasite sampling was obtained from the SODEPA in Cameroon (Société de Développement et d’Exploitation des Productions Agricoles). In Cameroon the majority of cattle breeding areas are situated in the north of the country (Fig. 1) and animals are transported to the main cities to be slaughtered. The sampling was done in the two main abattoirs situated in the south of Cameroon (i.e. Yaoundé and Douala). Before sampling, the stockbreeder was questioned to identify the provenance of the cattle. Only those which had their origin identified were included in the present study. In total, 480 cows (Table 1) originating from four distinct localities along a linear north-to-south transect in the eastern part of the country were checked for *S. bovis* infection (Fig. 1). Maroua is at the extreme north of the country and Garoua, Ngaoundéré and Bertoua are 175, 377 and 672 km south of Maroua, respectively.

**Table 1:** The number of cows inspected, the prevalence of schistosomiasis and the numbers of schistosomes recovered from the 22 cows sampled from the four areas of Cameroon included in the study

Origin of the cattle	No. of animals inspected	No. of positive animals	Prevalence of infection (%)	No. of animals sampled	No. of male/No. of female schistosomes
Ngaoundéré	70	23	13.5	7	36/34
Maroua	130	28	21.5	5	24/25
Bertoua	40	15	37.5	3	13/15
Garoua	140	28	20.0	7	38/33
Total	480	94	19.5	22	111/107

Upon slaughter, the mesenteric vessels of each cow were visually inspected for schistosome parasites. The observed adult schistosomes were recovered using forceps and washed using saline solution. Adult pairs were gently separated into males and females while alive before being individually stored in 95% ethanol for subsequent genetic analyses.

### Molecular methods

Total genomic DNA from a total of 218 individual schistosomes (111 males and 107 females) from 22 cows were individually extracted using the E.Z.N.A. tissue DNA extraction kit (Omega Bio-tek, Norcross, USA). All specimens were genotyped using a panel of microsatellites markers for population genetic analysis. A subsample of 91 individuals (46 males and 45 females) were further molecularly characterized using both the nuclear ITS and mitochondrial *cox*1 markers to clarify species and hybrid status and investigate mitochondrial genetic diversity. The details of this subsample is presented in Additional file 1: Table S1.

### Microsatellite genotyping

Individual samples were genotyped with a set of microsatellites initially developed for *S. haematobium* by Webster et al. [22]; however, *S. haematobium* and *S. bovis* are closely related sister species so the microsatellites were known to cross-react with *S. bovis* (Webster B, personal communication). Of the 18 microsatellites tested, 14 successfully amplified on our *S. bovis* samples and were thus used in the present study. The remaining four markers (C131, Sh7, Sh10 and Sh8) succeeded on less than 20% of the specimens and were thus discarded from our dataset. Microsatellite amplifications were performed using the Qiagen® Multiplex PCR kit, (Qiagen, Hilden, Germany). The forward primers were fluorescently labeled using 6-FAM, VIC, NED and PET dyes (Applied Biosystems, Foster City, USA) as per [22]. PCRs were carried out according to the manufacturer’s standard microsatellite amplification protocol except for the final volume of 10 μl including 2.5 μl of DNA template. Thermal cycling was performed with an initial hot-start activation of 15 min at 95 °C, followed by 30 cycles of 94 °C for 30 s, 56 °C for 90 s and 72 °C for 60 s, with a final extension at 60 °C for 30 min. Microsatellite reactions were performed using a TECNE TC-Plus Thermal Cycler (Cole-Palmer, Stone, UK). The microsatellite reactions were sent to Genoscreen (Lille, France) for genotype determination. Peak calling and genotype determination was done using GeneMarker Software. We used automatic size determination of the allele size using the Fragment Animal Analysis option of Genemarker Software with the GS500 size standard. All of the allele determination was double checked by visual inspection and the microsatellite matrix was exported as a data spreadsheet (Additional file 2: Table S2).

### *cox*1 and ITS amplification and sequencing

The partial *cox*1 and ITS gene regions were amplified from the DNA samples using primers defined by Lockyer et al. [23] and Barber et al. [24], respectively. PCRs were carried out in a final reaction volume of 25 μl, comprising 2 μl of DNA template, 5 μl 5× Colorless GoTaq® Flexi Buffer, 1.5 μl of MgCl_2_, 5 μl of dNTP (10 mM), 1 μl of each primer (10 μM) and 0.2 μl of Taq polymerase (Go taq®G2 Hot Start Polymerase). Cycling conditions were the same for both genes: 3 min at 95 °C followed by 35 cycles of 95 °C for 40 s, 52 °C for 40 s and 72 °C for 70 s, with an extension of 4 min at 72 °C. After amplification, 4.5 μl of the individual PCR products were stained with ethidium bromide, loaded on a 1.5% agarose gel and visually inspected to check the size and the quality of the amplicons (1100–1200 bp). All positive DNA products were then purified and sequenced in both directions (i.e. using both the forward and the reverse primers) on an Applied Biosystems Genetic Analyser at Genoscreen.

### Population structure and genetic diversity

#### Microsatellites

The genetic diversity of the schistosomes from each origin, defined as populations, was assessed by computing the expected heterozygosity (He), number of alleles (A), allelic richness (Ar) and Fis at each microsatellite locus using FSTAT v.2.9.3.2. [25]. These parameters were compared between the populations using the pairwise Friedman rank tests. The genetic differentiation between sites was assessed using pairwise Fst according to Weir & Cockerham [26] using FSTAT v.2.9.3.2. Next, we checked for potential genetic structure among individuals using a principal components analysis (PCA) as implemented in the Genetix software. We also performed an analysis of molecular variance (AMOVA) to evaluate the partitioning of the overall genetic variance among individuals according to three hierarchical levels: (i) within host; (ii) among hosts within the site of origin; and (iii) among sites. The AMOVA was performed using Arlequin software v.3.5 [27]. Finally, we determined the uppermost level of genetic structure of all individuals using the Bayesian clustering approach implemented in the Structure software [28]. In particular, we tested the number of clusters from K = 1 to K = 10. Three runs were computed for each K each of which consisted of 10^6^ iterations after a ‘burn-in’ period of 250,000 iterations, using an admixture model and the other parameters were set by default. The log likelihood for each K was then averaged over the three runs using the *corrsieve* package in R. The ΔK-values were then computed in R to determine the most likely K number among the K tested as described in Evanno et al. [29]. Two K-values (K = 2 and K = 4) were identified as the most likely number of genetic clusters. For each of these two K-values, an additional 10 runs were computed using 10^6^ iterations and setting the same initial parameters as those described previously. The probability for each individual to belong to each K was then averaged over the 10 runs (Q values). These Q values were then graphically represented using Clumpp [30] and the Distruct [31] software.

Finally, we took advantage of the fact that the microsatellite markers used in this study are informative for both *S. haematobium* and *S. bovis* to compare the genetic diversity between the two species based upon the 14 shared markers. To do so, we used a raw dataset from a previous article focusing on *S. haematobium* [22]. This raw dataset includes individual genotypes using 16 microsatellites (including the 14 markers used in this study) for 50 *S. haematobium* miracidia larvae from both Niger and Zanzibar [22]. Both, the allelic richness and the genetic diversity at each microsatellite locus were estimated using FSTAT v.2.9.3.2. for the two species and then compared using pairwise Wilcoxon rank tests.

#### ITS and cox1

The sequences were checked for possible sequencing errors and concatenated using Sequencher software v.4.5. The resulting cleaned sequences were next aligned using Bioedit v.7.0.9 and ClustalW software. Species assignation was done by comparing ITS and *cox*1 sequences to the GenBank nucleotide database. Concerning ITS, because this gene differs at 5 mutation points between *S. haematobium* and *S. bovis*, the sequence chromatograms were carefully checked at these mutation sites to identify possible heterogenous ITS sequences [32]. For the mitochondrial *cox*1 data, polymorphism indices (haplotype, nucleotide diversities, Tajima’s D* and Fu and Li’s D* statistics) were calculated using DnaSP v.6.0 software [33]. Significance of the genetic structuring patterns were tested using AMOVA [27]. The best-fit model of evolution (HKY+G) was determined using the maximum likelihood value implemented in MEGA v.6.0.6 [34]. A phylogenetic tree was constructed using MEGA v.6.0.6 software using a maximum likelihood method. The support for tree branching was based on 500 bootstrap replicates and rooted with *Schistosoma haematobium* (AY157209.1) as the outgroup. All sequences were uploaded to GenBank (accession numbers MH647122-MH647179).

## Results

### Parasitological survey

Among the 480 cows inspected, 94 were found to be positive (19.5%) for adult schistosome worms. Depending on the animals’ origin, the prevalence varied from 13.5 to 37.5% although there was no significant difference (*χ*^2^ = 4.8, *df* = 3, *P* = 0.18; Table 1).

### Microsatellite analysis

All the computed genetic diversity indexes (He, A, Ar and Fis) are presented in Table 2. No difference between localities was observed for He (*χ*^2^ = 4.036, *df* = 3, *P* = 0.258), Ar (*χ*^2^ = 2.66, *df* = 3, *P* = 0.45) and Fis (*χ*^2^ = 3.6, *df* = 3, *P* = 0.308). The only statistical difference was observed for the numbers of allele A (*χ*^2^ = 17.65, *df* = 3, *P* = 0.001), which could be attributed to the sampling size disequilibrium between the sites, from *n* = 28 in Bertoua to *n* = 71 samples in Garoua. The AMOVA analysis revealed that most of the genetic variation (98.09%) is found within animals and not between animals sampled (Table 3).

**Table 2:** Heterozygosity (He), number of alleles (A), allelic richness (Ar) and Fis for each microsattelite locus in each of the four schistosome populations studied. Allelic richness is based on a minimum of 24 diploid individuals

Loci	Ngaoundéré (*n* = 70)				Bertoua (*n* = 28)				Maroua (*n* = 49)				Garoua (*n* = 71)				All populations (*n* = 218)		
	He	A	Ar	Fis	He	A	Ar	Fis	He	A	Ar	Fis	He	A	Ar	Fis	He	A	Ar
Sh9	0.90	19	14.62	0.54	0.90	13	12.67	0.53	0.85	15	12.88	0.64	0.86	13	11.62	0.44	0.88	21	13.80
Sh3	0.94	22	17.82	0.00	0.95	20	19.33	-0.05	0.94	22	18.37	0.05	0.95	24	18.81	0.02	0.95	27	18.71
C102	0.53	9	6.24	0.24	0.35	3	2.86	0.19	0.54	7	5.69	0.34	0.49	6	4.18	0.19	0.48	11	5.41
Sh1	0.89	15	12.25	0.00	0.87	11	10.67	0.05	0.86	14	11.05	0.03	0.83	12	10.22	0.02	0.86	16	10.94
Sh14	0.83	15	10.76	0.00	0.86	13	12.10	0.21	0.77	13	10.06	0.02	0.83	15	10.99	0.09	0.82	21	10.74
Sh6	0.87	13	10.32	0.36	0.88	11	10.80	0.44	0.86	11	9.32	0.36	0.86	12	9.51	0.44	0.87	13	9.88
C111	0.49	4	2.97	0.63	0.55	3	3.00	0.39	0.48	5	4.19	0.46	0.47	2	2.00	0.36	0.50	6	3.15
Sh13	0.94	19	16.37	0.06	0.89	16	15.21	0.04	0.92	20	16.21	-0.02	0.93	20	15.92	-0.01	0.92	23	16.06
Sh4	0.92	18	14.76	0.07	0.90	11	10.83	0.08	0.92	16	14.01	0.00	0.92	18	14.26	0.02	0.91	19	14.23
Sh11	0.69	6	5.81	0.27	0.73	6	5.84	0.02	0.69	6	5.47	0.14	0.72	8	6.44	0.09	0.71	8	5.97
Sh15	0.25	3	2.83	0.11	0.34	3	3.00	0.05	0.26	3	2.87	0.12	0.32	3	2.82	0.09	0.29	3	2.84
Sh2	0.92	18	14.35	0.04	0.92	15	14.39	-0.09	0.92	17	14.68	-0.02	0.91	20	14.14	-0.05	0.92	23	14.19
Sh5	0.84	12	10.00	0.23	0.86	12	11.40	0.21	0.85	12	10.65	0.36	0.81	13	10.12	0.29	0.84	15	10.41
Sh12	0.60	5	4.23	0.19	0.51	3	3.00	0.29	0.55	3	3.00	0.37	0.56	5	3.88	0.36	0.56	6	3.83
Mean	0.76	12.71	10.24	0.20	0.75	10.00	9.65	0.17	0.74	11.71	9.89	0.20	0.75	12.21	9.64	0.17	0.75	15.14	10.01
SE	0.06	1.76	1.41	0.06	0.06	1.53	1.46	0.05	0.06	1.70	1.40	0.06	0.06	1.88	1.44	0.05	0.06	2.09	1.41

*Abbreviation*: *SE* standard error

**Table 3:** Analysis of the molecular variance within and among the *S. bovis* populations using *cox*1 and microsatellite data. The sum of squares is the sum of squared differences of each observation from the mean. Variance component estimates the contribution of the source of the variation to the variance

Genetic marker	*df*	Sum of squares	Variance components	Percentage of variation
*cox*1 analysis				
Among population	3	2.599	0.01147	2.36
Among animals within population	18	10.904	0.04276	8.81
Within animals	69	29.750	0.43116	88.83
Total	90	43.253	0.48538	
Microsatellite analysis				
Among populations	3	20.779	0.00239	0.05
Among animals within population	18	12.239	0.09253	1.86
Within animals	408	1987.705	4.87182	98.09
Total	429	2128.723	4.96674	

*Abbreviation*: *df* degrees of freedom

**Fig. 2:**
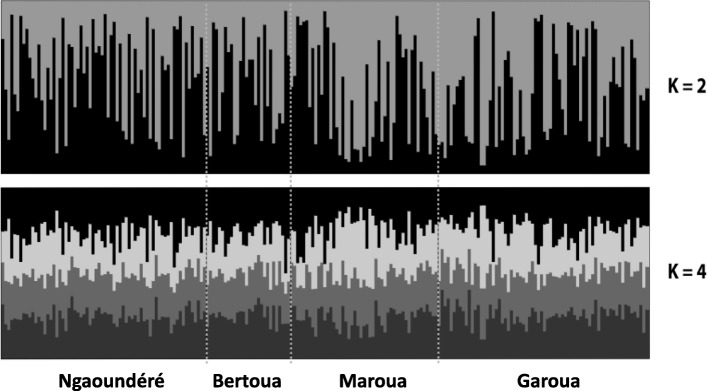
Structure bar plots obtained showing the lack of population structure of *S. bovis* from the 4 sampling areas in Cameroon for K = 2 and K = 4

Among the different K-values tested, K = 2 and K = 4 displayed maximal likelihood values suggesting that the individuals are grouped into 2 or 4 clusters (ΔK; Additional file 3: Figure S1). However, no apparent genetic structure was observed among the samples. Indeed, all individuals displayed a genetic background that could not have been clearly attributed to any individual genetic group (population) or to any of the sampling areas (Fig. 2).

**Fig. 3:**
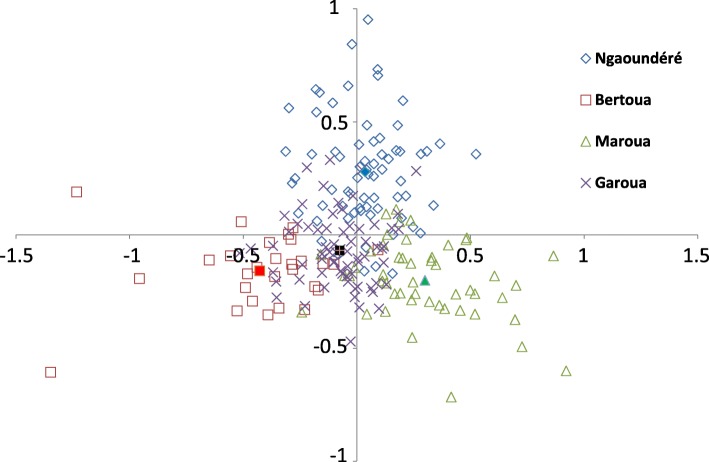
Principal components analysis (PCA) plot. Each parasite is represented by a dot and the color label corresponds to their population origin. A total of 28 variables (14 microsatellites × 2 alleles) were used in the analysis. The first and the second axis explained 38.77% and 32.29% of the genetic variation, respectively

The data showed that despite the large geographical scale of the sampled cows there is no, or negligible, genetic structure between the different schistosome populations. This is also illustrated by the pairwise Fst values computed between sites that do not exceed 0.5% (Table 4). Similarly, the first two axes of the PCA (accounting for 38.77 and 32.29% of the total variation for principal components 1 and 2, respectively) showed very weak structuration among the four populations sampled (Fig. 3).

**Table 4:** Pairwise estimates of Fst values (below the diagonal) of *S. bovis* parasite populations based on microsatellite DNA genotypes

	Ngaoundéré	Bertoua	Maroua
Ngaoundéré	**–**	ns	ns
Bertoua	0.0048	**–**	ns
Maroua	0.0012	0.0043	**–**
Garoua	0.0019	0.0002	0.0017

*Abbreviation*: *ns* no statistically significant differences at the *P* < 0.05 level

### Interspecific comparisons

Table 5 presents the allelic richness and gene diversity for *S. bovis* from this study and *S. haematobium* from [22]. The two *S. haematobium* populations (Niger and Zanzibar) presented both lower allelic richness and lower diversity than the whole *S. bovis* population from Cameroon. The difference was statistically significant for allelic richness only (Wilcoxon rank test, *P* < 0.05).

**Table 5:** Allelic richness and genetic diversity for *S. haematobium* and *S. bovis*. Indices for *S. haematobium* are from Webster et al. [22]. Indices for *S. bovis* are from the present study. Allelic richness is based on 50 diploid individuals

Locus	Allelic richness			Gene diversity (%)		
	*S. haematobium*		*S. bovis*	*S. haematobium*		*S. bovis*
	Niger	Zanzibar	Cameroon	Niger	Zanzibar	Cameroon
Sh9	6.0	9.0	16.90	77	87	88
Sh3	14.0	13.0	22.39	87	87	95
C102	5.0	2.0	7.26	37	2	50
Sh1	8.0	9.0	12.86	73	81	86
Sh14	11.0	13.0	13.89	86	89	83
Sh6	5.0	8.0	11.53	45	76	86
C111	6.0	6.0	4.05	68	69	49
Sh13	12.0	13.0	18.89	72	65	93
Sh4	9.0	9.0	16.59	79	80	92
Sh11	6.0	6.0	6.45	59	69	70
Sh15	5.0	7.0	2.98	66	47	28
Sh2	16.0	16.0	17.15	91	90	91
Sh5	12.0	7.0	12.32	82	48	83
Sh12	3.0	6.0	4.51	6	66	57
Mean	8.42	8.86	11.98	66	68	75
SE	1.06	1.0	1.63	6	6	6

*Abbreviation*: *SE* standard error

### Sequence analyses

Only one ITS genotype was identified among the 91 screened schistosomes and this was identified as *S. bovis*. No double peak was observed in any sequence at the polymorphic sites between *S. bovis* and *S. haematobium*. All the *cox*1 sequences were also identified as *S. bovis*. Thus, according to these two markers all the schistosomes analyzed were considered as pure *S. bovis*. A total of 58 *cox*1 haplotypes (accession numbers MH647122-MH647179) were identified. The haplotype diversity (± SD) and nucleotide diversity (± SD) were 0.961 ± 0.012 and 0.00602 ± 0.00043, respectively. No difference in haplotype and nucleotide diversity was observed according to the sampling site (Table 6). Both Tajima’s D* (-2.24; *P* < 0.01) and Fu and Li’s D* (-4.61; *P* < 0.02) statistics were significantly negative.

**Table 6:** Number of haplotypes, haplotype diversity and nucleotide diversity of the *cox*1 sequences from *S. bovis* sampled at the four sites in Cameroon

Area of origin	No. of haplotypes/no. of sequences	Haplotype diversity	Nucleotide diversity
Ngaoundéré	28/33	0.985 ± 0.014	0.00718 ± 0.00067
Maroua	12/20	0.847 ± 0.079	0.00407 ± 0.00077
Bertoua	9/12	0.909 ± 0.079	0.00451 ± 0.00129
Garoua	19/26	0.948 ± 0.034	0.00621 ± 0.00076
All	68/91	0.961 ± 0.012	0.00602 ± 0.00043

AMOVA analysis revealed that 88.83% of the genetic variation can be explained by the variation within animals whereas the remaining is due to variation among animals within sites (8.81%) or among sites (2.36%) (Table 3), which corroborates the results obtained from the microsatellite dataset. The absence of any genetic structuring with regard to the area of origin is confirmed by no or very low branch support and significant mixing of the populations on the phylogenetic tree (Additional file 4: Figure S2).

## Discussion

The four localities from which animals were screened in this study belong to the northern region of Cameroon which possesses 90% of Cameroon’s cattle herds (Fig. 1) [35]. Other intensive cattle breeding areas exist in the west and north-west regions; however, animals were not obtained from these regions. On average, we found that 19.5% of the inspected cows were infected (range 13.5–37.5%). This prevalence is much lower than that found by Ousseni in 1990 [15]. Such a difference might be at least partly explained by non-exclusive hypothetical factors including the slaughter season, or the age and welfare of the inspected animals. Indeed, all these parameters are known to influence infection prevalence in cows [36]. Unfortunately, we lack information to test these hypotheses.

The sequence data indicate that all the infections were of pure *S. bovis* and we did not find any evidence of interspecific hybridization, consistent with the fact that *S. haematobium* × *S. bovis* hybrids have yet to be found in cattle [32]. However, just like Webster et al. [32], we did not inspect the veins bordering the urinary bladder of the cows, and so any potential *S. haematobium* or hybrid schistosomes may have been missed. The presence of *S. haematobium × S. bovis* hybrids in humans have been suspected in the northern region of Cameroon [37]. These data were based on an ITS2-RFLP molecular approach to identify hybrid profiles between *S. haematobium* and other species from the *S. haematobium* group; however, the resolution of this method is not high enough to distinguish *S. bovis*, *S. mattheei*, *S. curassoni* and *S. guineensis*. The authors hypothesized that these hybrids were *S. haematobium × S. bovis* hybrids, but because *S. guineensis* is also present in Cameroon and the ITS is identical for *S. guineensis* and *S. bovis*, the nature of these hybrids remains unclear [10, 17].

Both the mitochondrial (*cox*1) and nuclear markers (microsatellites) indicated that most of the genetic variation observed among parasites is observed within and not between sampled animals. The factors influencing the genetic diversity of schistosomes were nicely depicted in the *S. mansoni-Rattus rattus* system on an island of Guadeloupe (West Indies) [38]. In particular, co-infections by multiple genotypes released by intermediate host snails, the dispersal ability of larval forms of different genotypes, and the movement/dispersion of the definitive host all result in important genetic diversity at the infrapopulation level. In this regard, it has been proposed that the definitive hosts act as “genetic mixing bowls” for the parasites [39]. Because this study is the first on *S. bovis*, only interspecific comparison with closely related species using relevant comparable markers can be performed. Previous studies using *cox*1 gene sequencing show a distinct pattern of genetic diversity according to the schistosome species concerned. At the scale of the African continent, the haplotype diversity (h) and the nucleotide diversity (Π) were 0.94 ± 0.0067 and 0.02553 [40] and 0.36 ± 0.014 and 0.00434 [41] for *S. mansoni* and *S. haematobium*, respectively. Thus the two parameters converge with *S. mansoni* having both higher haplotype and nucleotide diversity than *S. haematobium*. Our study shows that *S. bovis* is more polymorphic than its sister species *S. haematobium* (based on both nuclear and mitochondrial markers) but also shows that the haplotype diversity is similar to that of *S. mansoni* (h = 0.961 ± 0.012) while the nucleotide diversity does not significantly differ from that of *S. haematobium* (Π = 0.00602). Moreover, we found that the Tajima’s D* and Fu and Li’s D* computed on our *S. bovis* dataset were significantly negative, which could constitute a signature of population demographic expansion [42]. These results contrast with Tajima’s D* estimates recently computed on both *S. mansoni* and *S. haematobium* in Yemen (both estimates did not differ from zero [43]). This major demographic difference between schistosomes associated with cattle or humans might be explained by the absence of or very few anthelminthic treatments in animals. Indeed, the increasing pressure of praziquantel treatments fortunately strongly affects the demographic dynamics and the genetic composition of human schistosome populations [44, 45].

Whatever the method or the marker (mitochondrial or nuclear) used, we did not find any evidence for inter- or intrapopulation structuring despite considerable geographical distances separating the populations. This pattern clearly differs from that of *S. mansoni* for which populations are generally strongly genetically structured with high Fst values even at small geographical scales. Such strong genetic structuring has been evidenced in several countries including Kenya [46], Brazil [39], Uganda [47], Ethiopia [48] and Senegal [49]. Conversely, little is known on the genetic structuring of *S. haematobium* populations [50, 51]. Based on a phylogeographic approach performed at the African continent scale, Webster et al. [41] revealed that *S. haematobium* [52] is poorly structured compared to *S. mansoni* [51]. Several factors might explain the absence of genetic structure in *S. bovis* populations. First, as previously mentioned, the absence of anthelminthic treatments in cattle might foster the expansion of *S. bovis* populations. The effective population size for *S. bovis* is expected to be large enough to limit the effect of possible genetic drift and population differentiation. Secondly, host dispersal is known to promote parasite dispersion, which in turn might homogenize populations at the genetic level. Dispersion of *S. bovis* is mainly driven by cattle movements across the country through transhumance practices. Transhumance is a common practice where pastoralists move their livestock seasonally across different grazing lands [53]. While these movements are beneficial in terms of optimizing the use of land and other natural resources [54], they also may play a role in disease transmission [55] and have other significant regional or local social/economic impacts [56]. The specific movement patterns of animals in conjunction with environmental conditions (wet *versus* dry and cold *versus* hot) could result in significant changes in whether transmission of infectious diseases is prevented or facilitated by movements [57]. In Cameroon, transhumance occurs frequently between the drier northern and the humid southern regions. Indeed, livestock in this country are mainly assured by a group of pastoralists collectively called the Fulanis who practice transhumance as the pendular and seasonal movement of the herds and as their lifestyle. For that purpose, during the long dry seasons, these nomads move south with their herds to find more grazing sites and also for economic reasons. In fact, livestock farming in not routinely practiced in the south but is more common in the West and North West regions.

## Conclusions

This study provides novel insights into the population genetics of *S. bovis*. No strong genetic structure was observed at the country scale but some genetic indices could be associated as a signature of population demographic expansion. More studies on genetic diversity and population genetic structuring of animal schistosomes are needed to infer the role of the definitive hosts on parasite gene flow.

## Additional files

Additional file 1: **Table S1.** Details of the schistosomes from different cows that were molecularly characterized using the nuclear ITS and mitochondrial *cox*1 markers. (DOCX 13 kb)

Additional file 2: **Table S2.** Microsatellite dataset. (XLS 47 kb)

Additional file 3: **Figure S1.** ΔK-values calculated by Evanno’s method detecting K = 2 and K = 4 subpopulations as the most genetically probable within the 14 loci analyzed. (DOCX 32 kb)

Additional file 4: **Figure S2.** Mitochondrial *cox*1 maximum likelihood tree based of the *S. bovis* samples. The code for each sample corresponds to the sampling site-the animal number-the sex of the schistosome (M or F) and the parasite number. (DOCX 21 kb)

## Abbreviations

ITS: Internal transcribed spacer
*cox*1: Cytochrome *c* oxidase subunit 1
