# Supplementary material for: Population genetic structure of Schistosoma bovis in Cameroon
Source: Parasit Vectors. 2019 Jan 24;12:56. doi: 10.1186/s13071-019-3307-0 (PMC6346511; doi:10.1186/s13071-019-3307-0)
Supplement: Supplementary file 1 — Table S1. Details of the schistosomes from different cows that were molecularly characterized using the nuclear ITS and mitochondrial cox1 markers. (DOCX 13 kb) [file 13071_2019_3307_MOESM1_ESM.docx]

**Additional file 1: Table S1.** Details of the schistosomes from different cows that were molecularly characterized using the nuclear ITS and mitochondrial *cox*1 markers

| Area | Animal Code | Male Schistosomes | Female Schistosomes |
| --- | --- | --- | --- |
| Bertoua | A4 | 2 | 2 |
|  | A5 | 2 | 2 |
|  | A6 | 2 | 2 |
|  |  |  |  |
| Garoua | A10 | 2 | 2 |
|  | A11 | 2 | 2 |
|  | A12 | 2 | 2 |
|  | A20 | 2 | 2 |
|  | A21 | 2 | 2 |
|  | A22 | 1 | 2 |
|  | A9 | 2 | 1 |
|  |  |  |  |
| Maroua | A17 | 2 | 2 |
|  | A18 | 2 | 2 |
|  | A19 | 2 | 2 |
|  | A7 | 2 | 2 |
|  | A8 | 2 | 2 |
|  |  |  |  |
| Ngaoundere | A1 | 6 | 4 |
|  | A2 | 2 | 2 |
|  | A3 | 2 | 2 |
|  | A13 | 2 | 2 |
|  | A14 | 2 | 2 |
|  | A15 | 2 | 2 |
|  | A16 | 1 | 2 |
| Total |  | 46 | 45 |
