# Supplementary material for: Population genetic structure of Schistosoma bovis in Cameroon
Source: Parasit Vectors. 2019 Jan 24;12:56. doi: 10.1186/s13071-019-3307-0 (PMC6346511; doi:10.1186/s13071-019-3307-0)
Supplement: Supplementary file 3 — Figure S1. ΔK-values calculated by Evanno’s method detecting K = 2 and K = 4 subpopulations as the most genetically probable within the 14 loci analyzed. (DOCX 32 kb) [file 13071_2019_3307_MOESM3_ESM.docx]

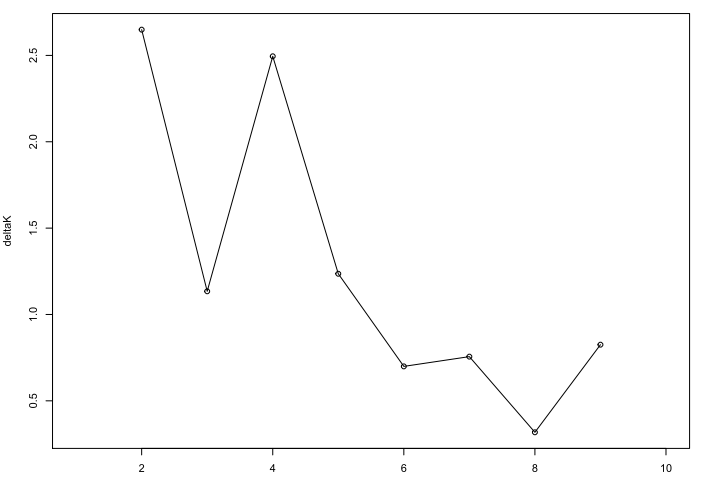


**Additional file 3: Figure S1.** ΔK-values calculated by Evanno’s method detecting K = 2 and K = 4 subpopulations as the most genetically probable within the 14 loci analyzed
