# Supplementary material for: Population genetic structure of Schistosoma bovis in Cameroon
Source: Parasit Vectors. 2019 Jan 24;12:56. doi: 10.1186/s13071-019-3307-0 (PMC6346511; doi:10.1186/s13071-019-3307-0)
Supplement: Supplementary file 4 — Figure S2. Mitochondrial cox1 maximum likelihood tree based of the S. bovis samples. The code for each sample corresponds to the sampling site-the animal number-the sex of the schistosome (M or F) and the parasite number. (DOCX 21 kb) [file 13071_2019_3307_MOESM4_ESM.docx]

|  |
| --- |

**Additional file 4: Figure S2.** Mitochondrial *cox*1 maximum likelihood tree based of the *S. bovis* samples. The code for each sample corresponds to the sampling site-the animal number-the sex of the schistosome (M or F) and the parasite number.
